# Assessment of the Rotation Motion at the Papillary Muscle Short-Axis Plane with Normal Subjects by Two-Dimensional Speckle Tracking Imaging: A Basic Clinical Study

**DOI:** 10.1371/journal.pone.0083071

**Published:** 2013-12-20

**Authors:** Xian-Da Ni, Jun Huang, Yuan-Ping Hu, Rui Xu, Wei-Yu Yang, Li-Ming Zhou

**Affiliations:** 1 Department of Ultrasound, The First Affiliated Hospital of Wenzhou Medical University, Wenzhou, China; 2 Department of Echocardiography, Changzhou No. 2 People’s Hospital Affiliated to Nanjing Medical University, Changzhou, China; 3 Department of Ultrasound, The First Affiliated Hospital of Henan university of TCM, Zhengzhou, China; 4 Department of Ultrasound, The second Affiliated Hospital of ZheJiang Univercity, Hangzhou, China; Semmelweis University, Hungary

## Abstract

**Background:**

The aim of this study was to observe the rotation patterns at the papillary muscle plane in the Left Ventricle(LV) with normal subjects using two-dimensional speckle tracking imaging(2D-STI).

**Methods:**

We acquired standard of the basal, the papillary muscle and the apical short-axis images of the LV in 64 subjects to estimate the LV rotation motion by 2D-STI. The rotational degrees at the papillary muscle short-axis plane were measured at 15 different time points in the analysis of two heart cycles.

**Results:**

There were counterclockwise rotation, clockwise rotation, and counterclockwise to clockwise rotation at the papillary muscle plane in the LV with normal subjects, respectively. The ROC analysis of the rotational degrees was performed at the papillary muscle short-axis plane at the peak LV torsion for predicting whether the turnaround point of twist to untwist motion pattern was located at the papillary muscle level. Sensitivity and specificity were 97% and 67%, respectively, with a cut-off value of 0.34°, and an area under the ROC curve of 0.8. At the peak LV torsion, there was no correlation between the rotational degrees at the papillary muscle short-axis plane and the LVEF in the normal subjects(r = 0.000, p = 0.998).

**Conclusions:**

In the study, we conclude that there were three rotation patterns at the papillary muscle short-axis levels, and the transition from basal clockwise rotation to apical counterclockwise rotation is located at the papillary muscle level.

## Introduction

The anatomy of normal myocardium consists of subendocardial, middle wall and subepicardial myocardial fibers, because of the different orientation of the ventricular muscle fibers they lead to contractions in different planes. Subendocardial and subepicardial longitudinally oriented fibres lead to longitudinal contraction, middle wall circumferentially oriented fibres lead to circumferential shortening and the oblique subendocardial and subepicardial fibres in between lead to LV rotation [1]. When a normal heart is observed in any short-axis plane, the twisting motion of the left ventricle can be described as “The wringing of a linen cloth to squeeze out water” [2]. When viewed from the apex, in systole, the LV apex rotates counterclockwise, whereas the base rotates clockwise [3]. However, when a normal heart is viewed in any long-axis plane, the motion can be described as shortening of its long axis and thickening of its walls, as though the base of the heart pushes itself towards the apex [4]. The wringing motion can give novel insight into LV systolic and diastolic function.

Two-dimensional speckle tracking imaging(2D-STI) has been introduced as a method for angle-independent quantification of LV strain, strain rate and LV twist. The speckles are the results of constructive and destructive interference of conventional gray-scale ultrasound images [5]. Tracking the unique speckle pattern from one frame to the next, the myocardial motion can be followed. At present, the research is mainly focused on the torsion or torsion velocity between the apex and the base of the heart [6]–[9]. Because of the rotation theory at the short-axis, we just consider: whether there is a plane, which is the transition from basal clockwise rotation to apical counterclockwise rotation. The papillary muscle are another anatomical landmark in the LV which excludes the mitral valve. Therefore, we phrased a specific hypothesis which is the transition plane from basal clockwise rotation to apical counterclockwise rotation is at the papillary muscle short-axis plane. In this study we sought to investigate the rotation motion at the papillary muscle short-axis plane, and to verify whether the turnaround point of twist to untwist motion pattern can be found at the papillary muscle short-axis plane. And according to the different rotation patterns at the papillary muscle short-axis plane, divide the LV into two parts at the papillary muscle plane, to research the torsion between the apex and the papillary muscle short-axis plane(Tor_A-P_), the torsion between the papillary muscle and the base short-axis plane(Tor_P-B_), respectively.

## Methods

### Study Population

The study group consisted of 64 healthy subjects (mean age 40±13 years, 37 men, mean age 41±15 years, 27 women, mean age 38±9 years ). All of the subjects chosen had no evidence of hypertension or diabetes mellitus. Electrocardiogram, echocardiogram and laboratory tests were normal along with a normal physical examination. The study was approved by the Human Subjects Committee of Wenzhou medical university. All of the volunteers in the study are consent about this experiment, and what we did to the participants in this study are obey with the “WORLD MEDICAL ASSOCIATION DECLARATION OF HELSINKI Ethical Principles for Medical Research Involving Human Subjects”. All of the consents were verbal, before the volunteers were included, we told them what we did to them, and we also told that there was no harm to them, and the ethics committees approved this consent procedure, so we just did.

### Conventional Two-Dimensional Doppler Echocardiography

64 healthy subjects underwent conventional echocardiography examination by GE-Vivid 7 Dimension. Left Atrial (LA) diameters, inter-ventricular septum (IVS) and LV posterior wall (LVPW) thickness at the end of diastole period were measured by M-mode echocardiography. LV end-systolic, end-diastolic volumes and LV Ejection Fraction (LVEF) were calculated by Simpson’s biplane method. The peak velocity during early diastole (E) and late diastole (A) velocity of the mitral valve were measured by pulsed-wave Doppler, and then the ratio E/A was calculated. The rotational degrees in papillary muscle short-axis plane were measured at 15 different time points in the analysis of two heart cycles, Onset of QRS wave, Mid-isovolumic contraction (IVC), aortic valve opening (AVO), 25% of ejection phase, 50% of ejection phase, 75% of ejection phase, aortic valve closure (AVC), mid-isovolumic relaxation (IVR), mitral valve opening (MVO), peak early diastole (E-peak) and end of early diastole (E-end), onset of atrial filling (A-onset) and peak atrial filling, Onset of QRS wave and AVO of the second heart cycle (AVO2) [10]–[11]. ECG leads were connected to each patient, and using a breath-hold technique, standard high frame rate (60–90/s) of the basal, the papillary muscle and the apical short-axis plane views of three consecutive cardiac cycles were stored for off-line analysis. Onset of QRS wave of the electrocardiogram is called the beginning of the LV contraction, the time between onset of QRS wave and AVO is called IVC, the time from AVO to AVC is called ejection period. The time between AVC and MVO is called IVR, the time from MVO to MVC is called the diastole period.

We defined the proper short-axis levels based on anatomical landmarks as follows [12]: to get the standard parasternal long-axis position, that is, the patient is positioned in the left lateral decubitus position and the transducer is placed in the left third or fourth intercostal space near to the sternum. The orientation marker of transducer is directed towards the patient’s right shoulder, to ensure that the LV and aorta were most inline with the mitral valve in the middle of the sector [13]. The parasternal short-axis views are obtained by 90 degrees clockwise rotating the transducer orientation mark towards the patient’s left shoulder from the standard parasternal long-axis view. By angling the transducer superiorly or inferiorly with the orientation marker staying in the same place, the heart in short axis at different levels can be demonstrated, and standard high frame rate of the basal, the papillary muscle and the apical short-axis planes views of three consecutive cardic cycles were stored for off-line analysis. We defined the proper short-axis levels based on anatomical landmarks as follows: the basal (mitral valve), the papillary muscle level (the middle of the anterolateral and the posteromedial papillary muscle) and the apical level (beneath the papillary muscle level, without visible papillary muscle) [6].

### Data Analysis for LV Rotation Behaviour

**Figure 1:**
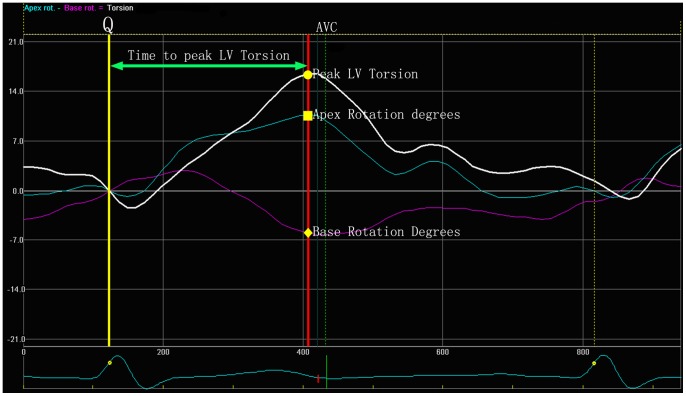
The yellow line means onset of QRS wave, the point of intersection of the red line and the white line means the peak LV torsion; The point of intersection of the red line and the purple line means the rotational degrees in the basal plane at the peak LV torsion; The point of intersection of the red line and the blue line means the rotational degrees in the apical plane at the peak LV torsion.

**Figure 2:**
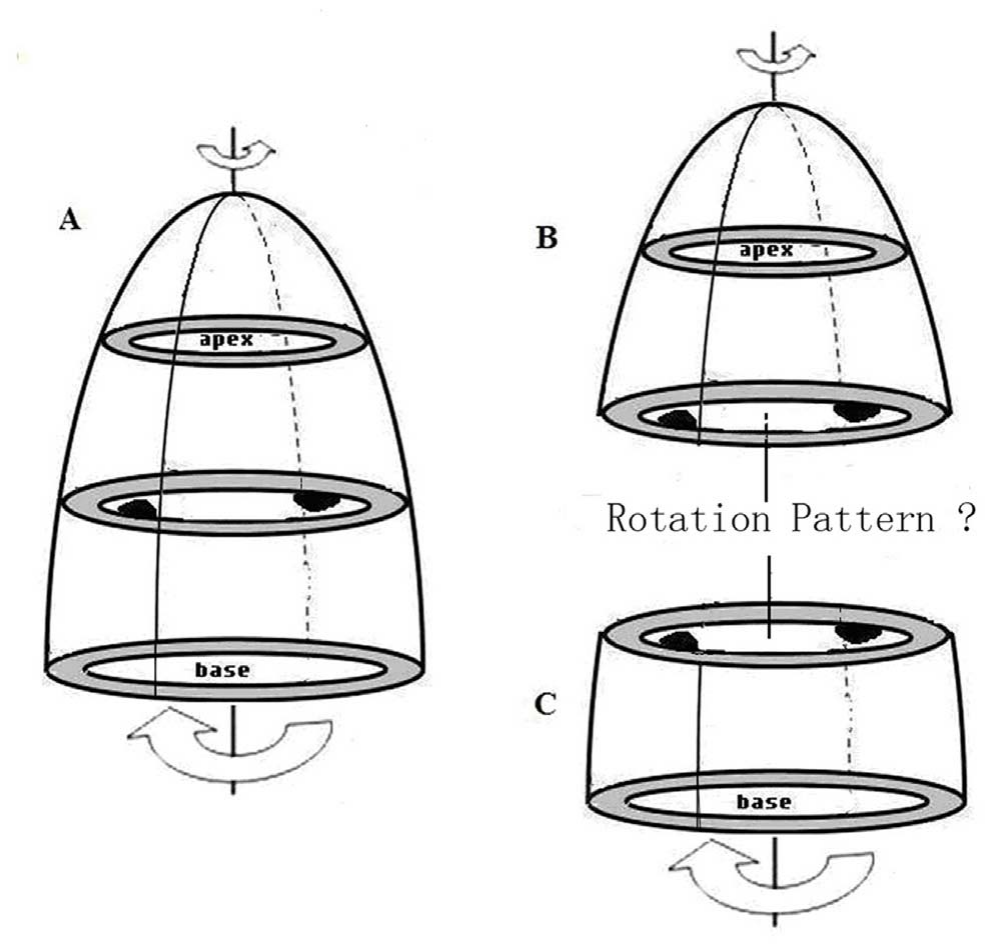
Divide the LV into two parts at the papillary muscle short-axis level.

We analysed the base, the papillary muscle and the apical short-axis levels views using 2D-STI software (2D-Strain, EchoPac PC v.7.x.x, GE Healthcare, Horten, Norway). Sketched the endocardial, then the software would automatic create a region of interest (ROI) which contained endocardial, middle layer and epicardial, Then adjusted the ROI in order to match the border of endocardial and epicardial well. The ROI was divided into six equally segments automatically (anteroseptal, anterior, lateral, posterior, inferior, septal), we measured the peak LV torsion using the following formula: LV torsion = apical LV rotation – basal LV rotation. We also recorded the time to the peak LV torsion; at the time to the peak LV torsion, we calculated the rotation degrees of the papillary muscle levels, the Tor_A-P_ and Tor_P-B_ in the peak LV torsion were calculated by the following formula: Tor_A-P_ = apical LV rotation – papillary muscle LV rotation, Tor_P-B_ = papillary muscle LV rotation – base LV rotation (Figure 1 and Figure 2). By convention, as viewed from the apex, counterclockwise LV rotation is expressed as a positive value and clockwise LV rotation as a negative one [3].

### Statistical Analysis

All of the analysis was performed using a commercially available package (SPSS13.0), According to the hypothesis, we defined the “counterclockwise to clockwise rotation pattern” at the papillary muscle short-axis plane as the normal pattern and considered the other rotation patterns as abnormal. The sensitivity and specificity of the rotational degrees at the papillary muscle short-axis plane at the peak LV torsion for predicting transition plane from basal clockwise rotation to apical counterclockwise rotation were derived by the receiver operating characteristic curve (ROC) analysis, We selected Youden’s index for the cut off value, which is defined by sensitivity + specificity − 1. At the peak LV torsion, Spearman’s correlation test was used for detecting the correlation between the rotational degrees at the papillary muscle short-axis plane and the LVEF in the normal subjects. Whether the data distribution was normal were assessed by Kolmogorov-Smirnov’s test. If the data distribution was normal, the data was compared with an independent student t-test; for variables with a non-normal distribution, the nonparametric Mann-Whitney test was used. To determine whether there were differences among the rotation patterns in the Tor_A-P_ or Tor_P-B_, one way ANOVA was used when appropriate. Data was expressed as the mean ± SD. Difference was considered statistically significant in all tests when the P value was less than 0.05.

## Results

**Figure 3:**
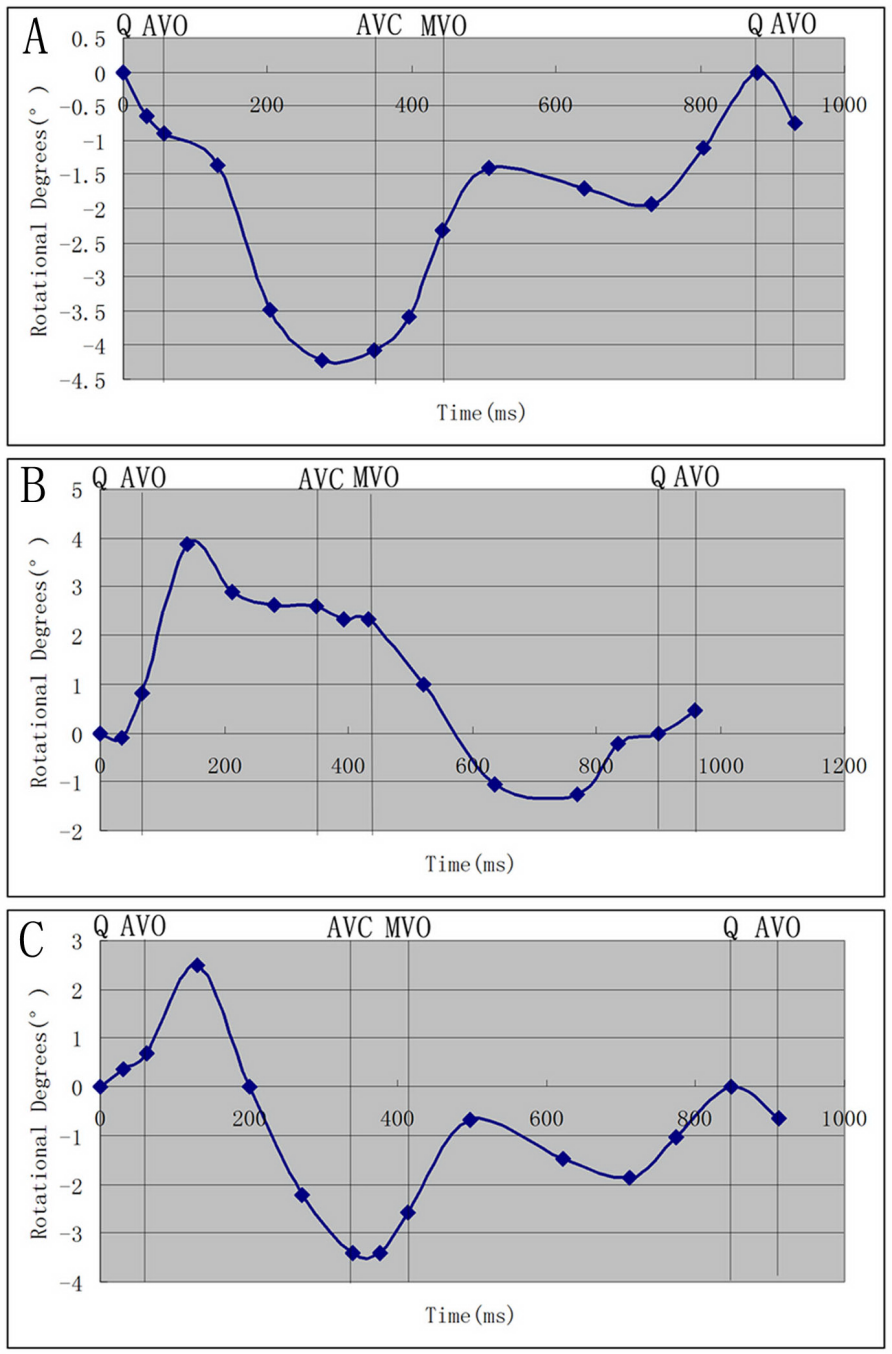
Three rotation patterns at the papillary muscle short-axis levels, **Figure 3A** is clockwise rotation, **Figure 3B** is counterclockwise rotation, and **Figure 3C** is counterclockwise to clockwise rotation.

**Figure 4:**
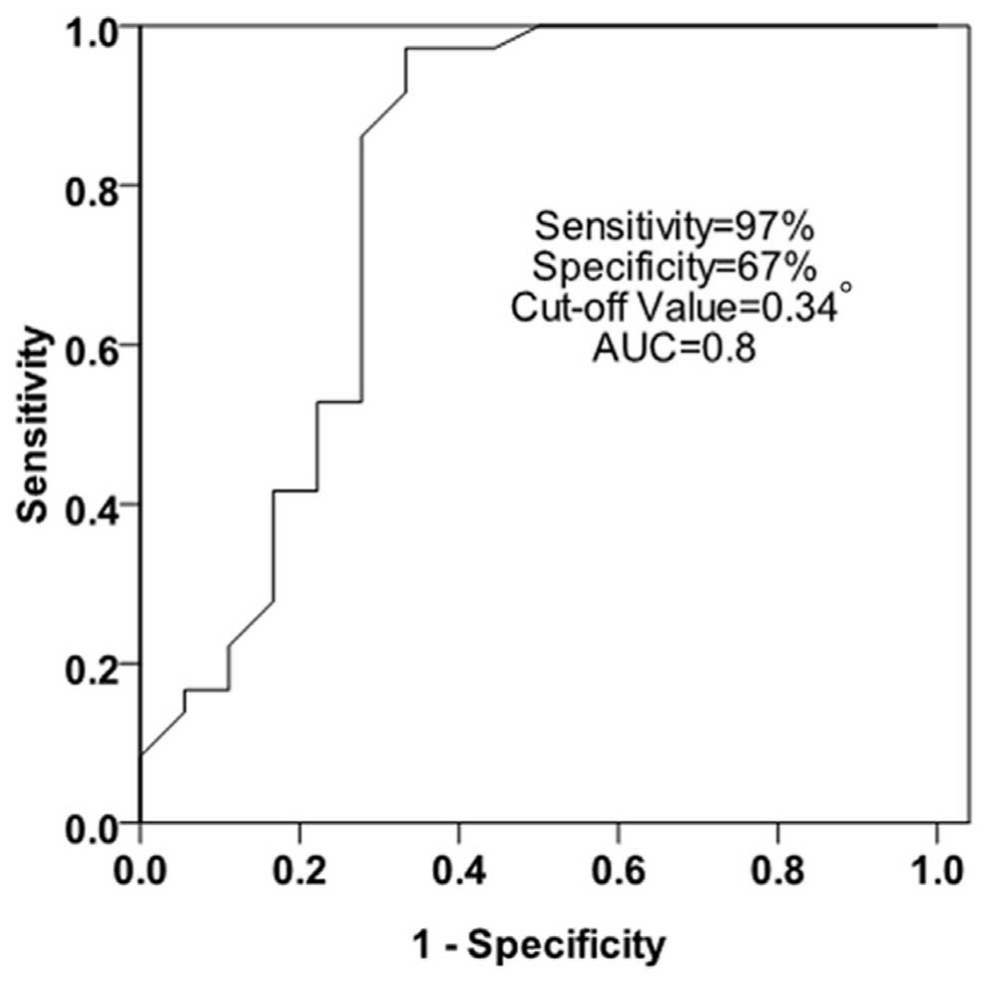
The ROC analysis of the rotational degrees was performed at the papillary muscle short-axis plane At the peak LV torsion for predicting whether the turnaround point of twist to untwist motion pattern was located at the papillary muscle level. Sensitivity and specificity were 97% and 67%, respectively, with a cut-off value of 0.34°, and an area under the ROC curve of 0.8.

**Figure 5:**
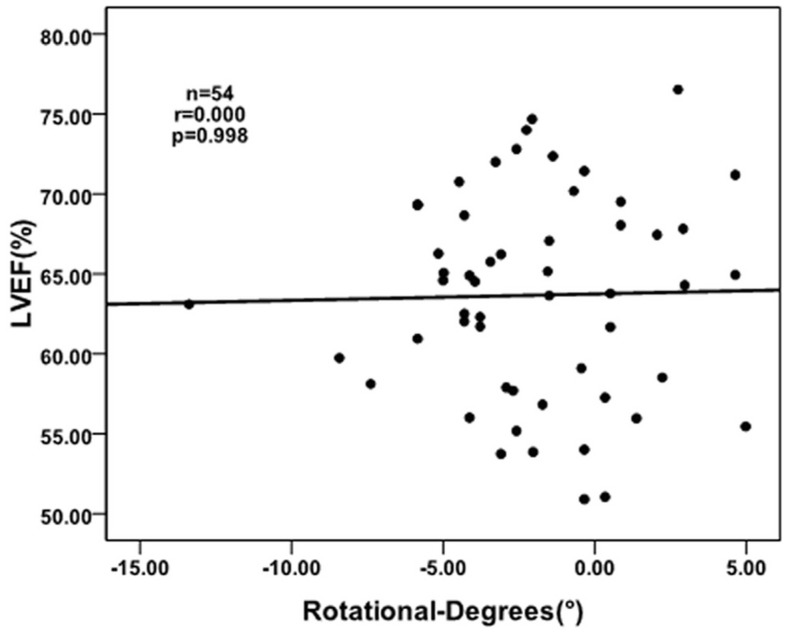
At the peak LV torsion, the correlation between the rotational degrees at the papillary muscle short-axis plane and the LVEF in the normal subjects (r = 0.000, p = 0.998).

1. In this study, 64 normal subjects were chosed for analysis. In 6 subjects, big differences in heart rate were insufficient for analysis. 2 subjects were excluded from analysis because the papillary muscle short-axis plane was unclear. In the remaining 56 subjects that made up the group about the papillary muscle short-axis plane rotation patterns. 2 subjects were excluded from analysis because the apical level was unclear, so there were only 54 subjects made up the peak LV torsion group. The diameters of LA, IVS, LVPW, the volume of LV end-systolic, the volume of LV end-diastolic, LVEF, E, A, and E/A ratio between men and women were recorded in the following form (Table 1).
2. As viewed from the LV apex, at the time to the peak LV torsion, the LV apex rotates countclockwise, whereas the LV base rotates clockwise. The rotational degrees in the base, the papillary muscle and the apical short-axis levels were −5.33°±3.66°, −1.99°±3.47°, 5.84°±3.78°, respectively.
3. Three rotation patterns at the papillary muscle short-axis levels were found in the systolic period. There are counterclockwise rotation (13 subjects), clockwise rotation (6 subjects) and counterclockwise to clockwise rotation (37 subjects), respectively (Table 2; Figure 3).
4. The ROC analysis of the rotational degrees was performed at the papillary muscle short-axis plane at the peak LV torsion for predicting whether the turnaround point of twist to untwist motion pattern was located at the papillary muscle level. Sensitivity and specificity were 97% and 67%, respectively, with a cut-off value of 0.34°, and an area under the ROC curve of 0.8 (Figure 4).
5. At the peak LV torsion, there was no correlation between the rotational degrees at the papillary muscle short-axis plane and the LVEF in the normal subjects (r = 0.000, p = 0.998) (Figure 5).
6. According to the three rotation patterns at the papillary muscle short-axis levels, to compare their torsion and their corresponding Tor_A-P_ and Tor_P-B_, respectively. When the papillary muscle short-axis levels rotate counterclockwise, there was no significant difference between Tor_A-P_ and Tor_P-B_ (5.43°±3.26°, 5.92°±4.02°, P>0.05), however, when the papillary muscle short-axis levels rotate clockwise or counterclockwise to clockwise, There were statistical differences between Tor_A-P_ and Tor_P-B_ (P<0.05), and the Tor_A-P_ was much larger than the Tor_P-B_ (Table 3).
7. In all of the normal subjects, at the time to the peak LV torsion, the statistical difference between Tor_A-P_ and Tor_P-B_ was significant (P<0.05), and the Tor_A-P_ was much larger than the Tor_P-B_ (7.83°±4.31°, 1.99°±3.47°, P<0.01) (Table 3).

**Table 1:** The basic Information of conventional two-dimensional Doppler echocardiography (mean ± SD).

Variable	All Subjects (64)	Men (37)	Women (27)	P-Value
Age (years)	40±13	41±15	38±9	NS
Heart Rate(bpm)	74±11	72±9	77±12	NS
LAD (mm)	34.48±4.14	35.51±3.52	33.07±4.57	P<0.05
LVEDV (ml)	82.02±19.27	89.43±19.25	71.85±14.79	P<0.01
LVESV (ml)	29.56±8.57	32.35±8.85	25.74±6.56	P<0.01
LVEF (%)	63.92±6.00	63.67±6.46	64.27±5.40	NS
IVS (mm)	6.16±1.00	9.59±0.80	8.63±1.11	P<0.01
LVPW (mm)	9.12±1.06	9.59±0.80	8.44±1.01	P<0.01
E (m/s)	0.87±0.15	0.84±0.16	0.91±0.13	P<0.05
A (m/s)	0.62±0.16	0.64±0.13	0.65±0.20	P<0.05
E/A	1.42±0.40	1.36±0.37	1.51±0.42	NS

**Table 2:** Three rotation patterns at the papillary muscle short-axis levels (mean ± SD).

Periods	Clockwise rotation		Counterclockwise rotation		Counterclockwise-clockwise rotation	
	Time (ms)	Total (°)	Time (ms)	Total (°)	Time (ms)	Total (°)
Q	0	0	0	0	0	0
IVS	31.33±5.65	−0.64±0.95	34.46±6.63	−0.09±0.70	31.76±7.69	0.36±2.28
AVO	57.17±11.81	−0.89±0.53	68.31±13.10	0.81±1.44	62.22±13.71	0.70±0.88
25%AVO	129.83±12.95	−1.36±0.86	140.31±15.64	3.88±2.27	131.19±15.09	2.50±1.53
50%AVO	202.50±16.07	−3.49±1.59	212.77±19.15	2.89±2.30	200.54±18.02	0.00±2.12
75%AVO	275.17±20.27	−4.22±2.04	281.46±23.73	2.63±1.78	270.59±22.01	−2.23±2.48
AVC	348.33±24.16	−4.08±2.55	349.23±26.27	2.60±1.56	338.81±26.70	−3.41±2.84
IVR	396.00±22.02	−3.59±2.94	392.46±31.17	2.33±2.02	376.30±30.49	−3.42±2.81
MVO	442.33±28.54	−2.32±3.42	432.31±38.12	2.32±2.33	412.68±37.48	−2.57±2.69
E-peak	507.17±29.03	−1.41±2.49	521.15±36.00	1.00±1.95	497.78±38.27	−0.67±2.53
E-end	639.17±39.10	−1.70±1.99	637.08±58.01	−1.05±1.59	622.41±58.03	−1.48±1.74
A-onset	733.00±108.92	−1.93±2.03	769.08±122.67	−1.26±1.18	711.05±113.21	−1.86±1.44
A-end	805.00±87.95	−1.11±1.39	834.46±117.67	−0.21±1.23	774.76±114.27	−1.02±1.40
Q-2	880.00±104.98	0	899.46±116.75	0	847.54±132.55	0
AVO-2	931.17±99.03	−0.75±0.52	959.23±121.86	0.47±1.63	911.43±133.03	−0.66±1.08

When viewed from the above values, positive values were considered as countclockwise rotation, while negative values were considered as clockwise rotation.

**Table 3:** Divide the LV into two parts at the papillary muscle short-axis levels, to compare their related parameters (mean ± SD).

Variable	Total (°)	counterclockwise (°)	clockwise (°)	Counterclockwise- clockwise (°)	F
Tor_A-P_	7.83±4.31	5.43±3.26	8.82±4.56	7.07±2.15	3.307^#^
Tor_P-B_	1.99±3.47**	5.92±4.02	2.65±3.61**	2.35±3.20*	3.954^#^
Peak Torsion	11.17±5.28	11.35±4.77	11.47±5.60	9.42±3.77	0.717

_A-P_ and Tor_P-B_ in the same rotation pattern, **means P<0.01, *means P<0.05. 1. When compare the values of Tor

_A-P_, Tor_P-B_, and Peak LV Torsion with different rotation patterns at the papillary muscle short-axis levels, ^#^means P<0.05. 2. When compare the values of Tor

“F” means the value of Analysis of Variance (ANOVA). 3.

### Reproducibility

Interobserver measurement of the rotational degrees at the papillary muscle short-axis plane At the peak LV torsion was determined by having a second investigator measure all chosen subjects, and the value was −2.10°±3.08°. For intraobserver variability, all subjects were analyzed twice by one investigator within an interval of one month, and the value was −1.91°±3.22°, the second intraobserver measurements were “blinded” to results from the initial measurements.

## Discussion

Due to the different orientations of the ventricular muscle fibers, results in a normal heart twist in the systolic period, and untwist in the diastole period, as viewed from the apex, in systole, the LV apex rotates counterclockwise, whereas the base rotates clockwise, and the results are consistent with previous studies. The subendocardial fibres were arranged in a right-handed helix and the subepicardial fibres in a left-handed helix. During the ejection period, although the subendocardial forces exceed subepicardial forces, the direction of rotation was dominated by subepicardial because of its larger radius. The large subepicardial torque resulted in global counterclockwise rotation near the LV apex and clockwise rotation at the LV base [1]–[2].

Previous studies about how papillary muscle short-axis level rotated have not been validated, Ulf Gustafsson, et al [10] thought that at the papillary muscle level there was a heterogeneous rotation with mainly clockwise rotation of the inferoseptal segments, whereas the anterolateral segments rotate counterclockwise, they indicated that the transition from basal clockwise rotation to apical counterclockwise rotation was located approximately at the middle papillary muscle level. Lu jing, et al [14]–[15] thought the rotation pattern of the papillary muscle level could be explained by the theory of a cylinder, which could be described as this: rotating at the two sides of a cylinder with the identical and the reverse power respectively, the middle position of the cylinder was relatively static. Due to the special anatomic structure of the LV, whether the papillary muscle level is in the mid-ventricle is needed to be considered. So the first aim of this research was to perform the rotation motion at the papillary muscle level, three rotation patterns at the papillary muscle short-axis levels were found in the systolic period. There were counterclockwise rotation (13 subjects), clockwise rotation (6 subjects) and counterclockwise to clockwise rotation (37 subjects), respectively.

**Figure 6:**
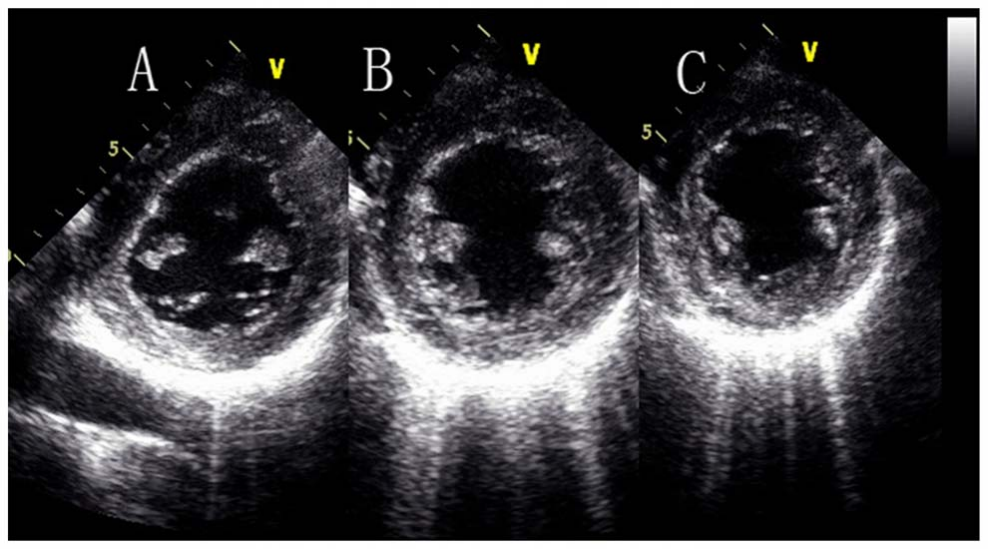
The papillary muscle short-axis levels at different locations. Figure 6A is a litter higher than the papillary muscle level but contain the papillary muscle, Figure 6B is in the middle of the papillary muscle level and Figure 6C is a litter lower than the papillary muscle level but contain the papillary muscle.

How did this phenomenon happen? There were two reasons may explained reasonable: firstly, according to the theory of “muscle band” [16]–[17], the band is oriented spatially as a helix formed by the basal and apical loops. The myocardial fibres in the basal loop are circumferential, while in the apical loop they are longitudinal. If the papillary muscle level is near to the basal loop, it may rotate clockwise, if near to the apical loop, it may rotate counterclockwise, and if the level is in the middle of the basal loop and the apical loop, the rotation pattern may be counterclockwise to clockwise [18]. Secondly, the rotation pattern at the papillary muscle level maybe related to the location of the papillary muscle. Because there is no generally accepted anatomical definition for the middle of papillary muscle level (the anterolateral and the posteromedial papillary muscle), we used the visual estimation to judge if the short-axis plane we stored is at the papillary muscle level. If the image we stored was not in the middle of the anterolateral and the posteromedial papillary muscle, just a little higher or lower than the papillary muscle level but contain the papillary muscle, this may produce the errors. We called this visual error, and it can not be avoid, we just do our best to make the image which we acquired in the middle of the anterolateral and the posteromedial papillary muscle, This maybe the another reason that caught the papillary muscle short-axis plane rotated in the three different patterns. (Figure 6).

The receiver operating characteristic curve (ROC) analysis of the rotational degrees was performed at the papillary muscle short-axis plane at the peak LV torsion for predicting whether the turnaround point of twist to untwist motion pattern was located at the papillary muscle level. Sensitivity and specificity were 97% and 67%, respectively, for the rotational degrees at the papillary muscle short-axis plane to predict the transition plane from basal clockwise rotation to apical counterclockwise rotation, with a cut-off value of 0.34°, and an area under the ROC curve of 0.8. These results showed that the transition from basal clockwise rotation to apical counterclockwise rotation short-axis plane is at the papillary muscle short-axis plane.

At the peak LV torsion, there was no correlation between the rotational degrees at the papillary muscle short-axis plane and the LVEF in the normal subjects (r = 0.000, p = 0.998), which may indicate that in the normal subjects, the rotational patterns at the papillary muscle short-axis plane do not influence the global systolic function of the LV, and the rotational degrees at the papillary muscle short-axis plane at the peak LV torsion are smaller than that in the apex and the base. It need further studies to determine the rotation patterns in patients with cardiovascular diseases, such as hypertension, dilated cardiomyopathy and hypertrophic cardiomyopathy, especially for those with abnormal LV function.

The second aim of this research, according to the three rotation patterns at the papillary muscle short-axis levels, was to compare their torsions and their corresponding Tor_A-P_ and Tor_P-B_, respectively. When the papillary muscle short-axis levels rotate counterclockwise, there was no significant difference between Tor_A-P_ and Tor_P-B_ (5.43°±3.26°, 5.92°±4.02°, P>0.05). This may be explained as this: at the time to the peak LV torsion, the papillary muscle level rotated the same direction as the apical level did. So the result in Tor_A-P_ became smaller, while the Tor_P-B_ became bigger. when the papillary muscle short-axis levels rotate clockwise or counterclockwise to clockwise, There were statistical differences between Tor_A-P_ and Tor_P-B_ (P<0.05), and the Tor_A-P_ was much larger than the Tor_P-B_. At the time to the peak LV torsion, the papillary muscle level rotated the same direction with the basal level, so the result in Tor_A-P_ became larger, and the Tor_P-B_ became smaller.

In all of the normal subjects, at the time to the peak LV torsion, the difference between Tor_A-P_ and Tor_P-B_ was significant (P<0.05), and the Tor_A-P_ was much larger than the Tor_P-B_ (7.83°±4.31°, 1.99°±3.47°, P<0.01), and there were 37 healthy subjects (66%) rotating from counterclockwise to clockwise so that we indicate the transition from basal clockwise rotation to apical counterclockwise rotation is located at the papillary muscle short-axis level.

## Conclusions

In this study, we conclude that there were three rotation patterns at the papillary muscle short-axis levels, and the transition from basal clockwise rotation to apical counterclockwise rotation is located approximately at the papillary muscle short-axis level. when we compared their torsions at the papillary muscle short-axis levels, we found that Tor_A-P_ was larger than Tor_P-B_.

### Clinical Implications

Although there is no correlation between the rotational degrees at the papillary muscle level at the peak LV torsion and the LVEF in the normal subjects, there is a need for further studies on the rotation patterns in patients with heart diseases, and the micro-changes in the rotation patterns at the papillary muscle levels may be a prediction factor in clinic diagnosis and treatment of some cardiovascular diseases.

### Limitations

Firstly, the limitations of 2D-STI is that the longitudinal motion of the LV causes the myocardium in the LV short-axis to move in and out of the image plane, especially at the LV base [19] (As the long axis shortens, the base of the heart pushes itself towards the apex, whereas the apex is relatively stationary). When the speckles tend to move out of plane during contraction, they cannot be tracked successfully by the software of Echopac. In order to make the data more accurately, we used the frame rate of 60–90/s and took a hold-breath technique. 3D speckle tracking image using real-time 3D data acquisition which has been introduced recently can limit the out of plane motion [20]. Secondly, if the normal subject is overweight, the intercostal space is narrow, or the subject has some problems within the lung, the images produced will be unclear for the software to analysis. Also large differences in heart rate were insufficient for analysis.
